# Biphenotypic sinonasal sarcoma with *PAX3::MAML3* fusion transforming into high-grade rhabdomyosarcoma: report of an emerging rare phenomenon

**DOI:** 10.1007/s00428-023-03501-0

**Published:** 2023-01-31

**Authors:** Anders Meyer, Natálie Klubíčková, Elaheh Mosaieby, Petr Grossmann, Antonina Kalmykova, Olena Koshyk, Michael Michal

**Affiliations:** 1 Department of Pathology, University of Kansas, KS Kansas City, USA; 2 Department of Pathology, Faculty of Medicine, Charles University, Medical Faculty and Charles University Hospital Plzen, Alej Svobody 80, 323 00, Plzen, Czech Republic; 3 Bioptical Laboratory, Ltd., Plzen, Czech Republic; 4 Medical Laboratory CSD Health Care, Ltd., Kyiv, Ukraine

**Keywords:** Biphenotypic sinonasal sarcoma, Rhabdomyosarcoma, High-grade transformation, PAX3::MAML3

## Abstract

We report a case of a 67-year-old male patient with a sinonasal tumor that showed areas of classic biphenotypic sinonasal sarcoma (BSNS) which in some sections sharply transitioned into high-grade rhabdomyosarcoma. Immunohistochemically, the conventional BSNS parts showed S100 protein, SMA, PAX7, and focal MyoD1 expression, whereas desmin and myogenin were negative. In contrast, the cells in high-grade areas expressed desmin, MyoD1, myogenin, and PAX7, while being negative for S100 protein and SMA. Using the Archer FusionPlex assay, the classical *PAX3::MAML3* gene fusion was detected. FISH for *PAX3* and *MAML3* confirmed a break of these genes in both components. Despite aggressive therapy, the tumor progression resulted in the patient’s death. The herein presented case, together with 2 previously published cases of BSNS with high-grade transformation, helps to better understand this novel phenomenon. Although the risk for such transformation appears low, it has important clinical and diagnostic implications which are discussed.

## Introduction

Biphenotypic sinonasal sarcoma (BSNS) was initially described by Lewis et al. in 2012 as a low-grade sarcoma with neural and myogenic features [1]. Clinically, these tumors usually follow an indolent course with frequent recurrences but no reported metastases and only exceptional disease-related mortality [1–4]. Morphologically, BSNS typically consists of an infiltrative hypercellular proliferation of uniform spindle cells arranged in fascicles, often with a herringbone pattern and frequent invaginations of hyperplastic surface mucosa. Mitotic activity is usually minimal. The vast majority of cases co-express smooth muscle actin (SMA) and S100 protein, while a minority of cases may also exhibit morphological and/or immunohistochemical signs of skeletal muscle differentiation [1, 3–5]. Fusions of *PAX3* gene are the molecular hallmark of BSNS, with *MAML3* being the fusion partner in the majority of cases [3, 4, 6]. Herein, we present a unique case of high-grade rhabdomyosarcoma (RMS) emerging from a typical BSNS with an aggressive clinical course.

## Case presentation

**Fig. 1:**
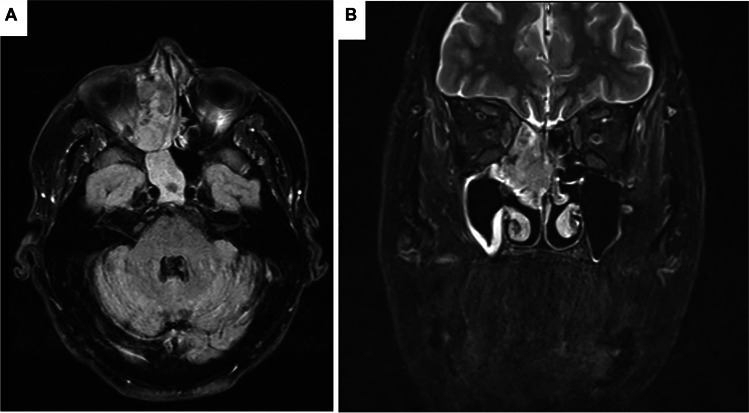
MRI on axial flair (**A**) and on coronal T2 short tau inversion recovery (**B**) showed a soft tissue mass involving the right ethmoid, maxillary, and frontal sinuses and invading the extraconal orbit causing mild asymmetric right-sided ocular proptosis

The patient was a 67-year-old male with Parkinson’s disease and a history of septoplasty, bilateral frontal sinusotomy, and removal of right middle turbinate concha bullosa 3 years before presenting with nasal congestion and epiphora. MRI showed a soft tissue mass involving the right ethmoid, maxillary, and frontal sinuses and invading the extraconal orbit causing mild asymmetric right-sided ocular proptosis (Fig. 1A,B). Endoscopic evaluation revealed a soft tissue mass measuring 4.4 × 3.4 × 2.2 cm which obscured the nasal airway and centered around the middle turbinate. Biopsy revealed a high-grade sarcoma with myogenic differentiation by immunohistochemistry, diagnosed as embryonal RMS. Staging PET was negative for metastatic disease. The patient initiated neoadjuvant chemotherapy with vincristine-dactinomycin-cytoxan. Repeated imaging after three cycles showed local progression and increased FDG avidity without metastatic disease.

**Fig. 2:**
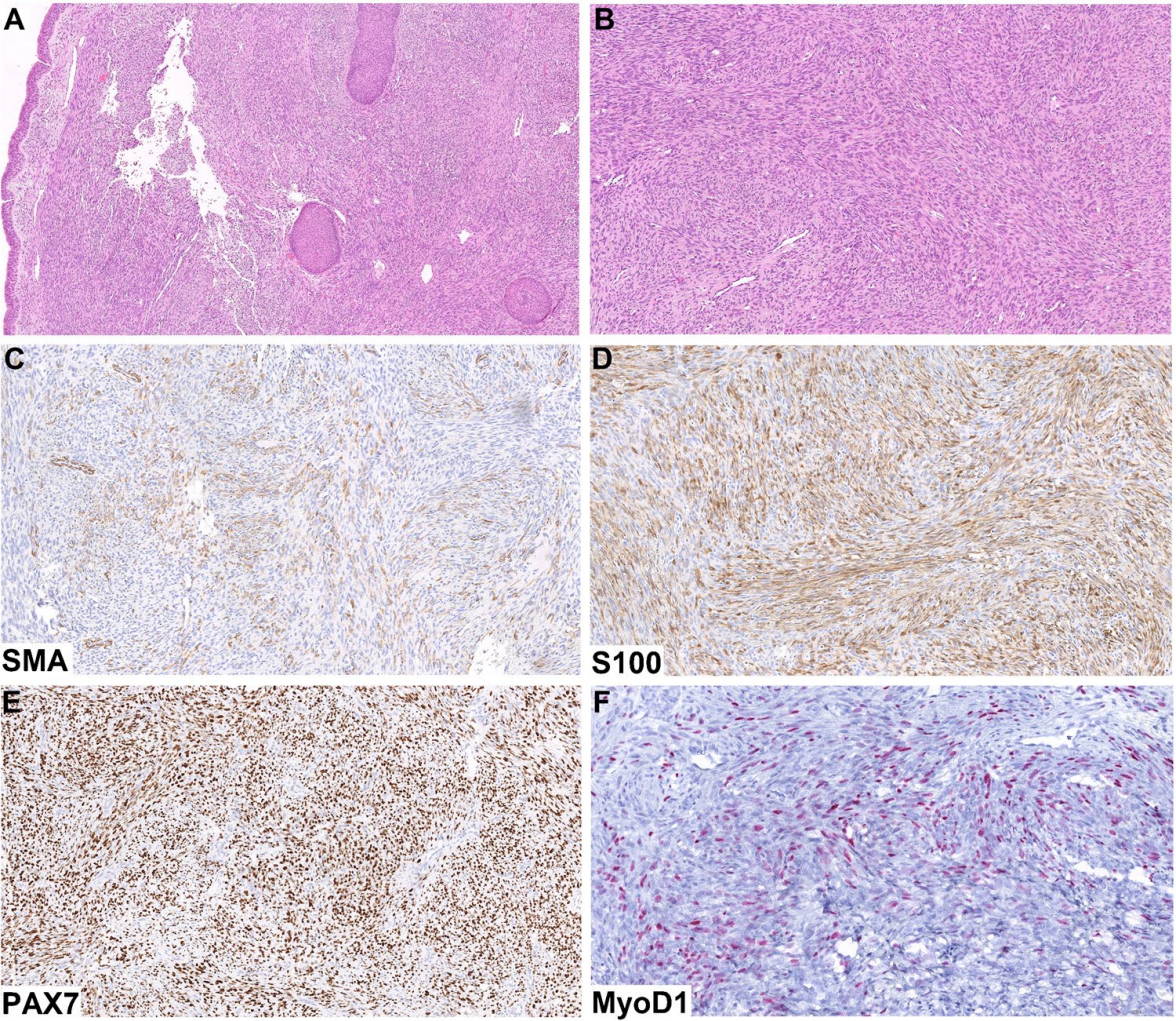
The resection specimen showed a proliferation of uniform spindled cells with moderate to high cellularity, occasional fascicular arrangement, minimal atypia and no mitotic activity (**B**). In some areas, invaginations of the hyperplastic surface mucosa were enveloped by the tumor cells (**A**). Immunohistochemically, the conventional BSNS areas showed patchy SMA (**C**) and diffuse S100 protein (**D**) expression. There was also a diffuse strong positivity with PAX7 (**E**) and patchy expression of MyoD1 (**F**)

**Fig. 3:**
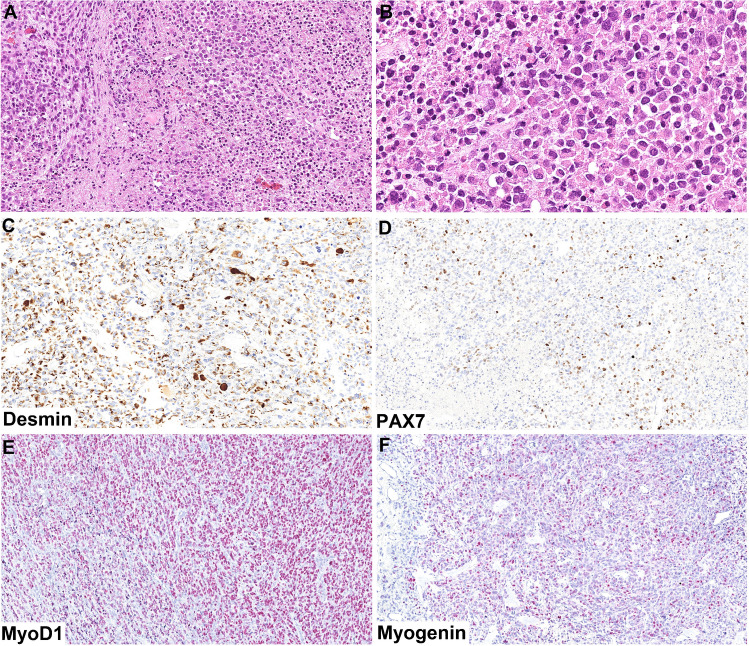
In some sections, this typical BSNS morphology sharply transitioned to a high-grade sarcoma with rhabdoid features, very high mitotic activity, and areas of necrosis (**A, B**). There was only patchy positivity with PAX7 (**D**), whereas the expression of desmin (**C**), MyoD1 (**E**), and myogenin (**F**) was diffused in these parts

The resection specimen showed a proliferation of uniform spindled cells with moderate to high cellularity, occasional fascicular arrangement, minimal atypia, and no mitotic activity (Fig. 2B). In some areas, invaginations of the hyperplastic surface mucosa were enveloped by the tumor cells (Fig. 2A). No rhabdoid differentiation was noted by morphological examination in this area. However, in some sections, this typical BSNS morphology sharply transitioned to a high-grade sarcoma with rhabdoid features, very high mitotic activity, and areas of necrosis (Fig. 3A,B).

Immunohistochemically, the conventional BSNS areas showed patchy SMA (Fig. 2C) and diffuse S100 protein (Fig. 2D) expression. There was also a diffuse strong positivity with PAX7 (Fig. 2E) and patchy expression of MyoD1 (Fig. 2F), while desmin and myogenin were completely negative. In contrast, the high-grade areas were completely negative for S100 protein and SMA, and there was only patchy positivity with PAX7 (Fig. 3D), whereas the expression of desmin (Fig. 3C), MyoD1 (Fig. 3E), and myogenin (Fig. 3F) was diffuse in these parts.

Based on the presence of the typical BSNS areas, molecular studies were performed using Archer FusionPlex assay as described previously [7]. This analysis revealed *PAX3*(exon7)*::MAML3*(exon2) fusion which was confirmed by FISH, using *MAML3* (4q31.1 ) and *PAX3* (2q36.1) break-apart probes (both from SureFISH, Agilent). Of note, the FISH analysis confirmed the presence of the rearrangement (with a cut-off defined as at least 10% cells with a break) in both the conventional and high-grade components.

Postoperatively, the patient received 5040 cGy in 28 fractions. Imaging studies 4 months after resection demonstrated recurrence along the right medial orbital wall and orbital floor. He was initiated on vincristine-urinotecan-temozolamide. Imaging after three cycles again showed local progression. Clinically, the tumor caused total vision loss in the right eye and started to protrude from the right nare. Due to continued progression on maximal therapy, the patient transitioned to hospice and died 15 months after his initial diagnosis.

## Discussion

BSNS with morphologically evident rhabdomyoblastic differentiation has been described in approximately 10% of cases [1–3, 5]. However, as the 2 largest studies have shown, at least focal immunohistochemical expression of desmin or MyoD1 is common, with the percentage of immunopositive cases ranging from 35 to 66% and 26 to 91%, respectively. Myogenin expression is the least frequent and is encountered in only 20% of cases [3, 4]. PAX7 expression in BSNS has not been extensively studied but was noted previously as well [8]. The skeletal muscle phenotype of BSNS is in line with its molecular background, which is most commonly characterized by fusions of *PAX3* with either *MAML3* or (less commonly) with *FOXO1* or *NCOA1*. Very rarely, other fusions partners may be involved, including *RREB::MRTFB* (*MKL2*) [3–6, 9–11]. However, the nosological classification of the latter as BSNS is somewhat controversial as identical gene fusions have been described in other head and neck mesenchymal tumors [10–12]. During development, PAX3 determines the cell fate of melanocytic, neuronal, and skeletal muscle differentiation and regulates normal myogenesis and postnatal muscular regeneration [5, 6, 13], while MAML3 has been shown to function as a potent transactivator of PAX3 response elements [6]. Gene expression profiling of BSNS with *PAX3::MAML3* fusion also showed altered expression of several genes and signaling networks involved in neural crest, skeletal system, and general embryonic development, including the myogenic genes *MYOCD* and *MYOD1* [6]. Interestingly, the *PAX3::FOXO1* and *PAX3::NCOA1* fusions were also described in rare cases of alveolar RMS [5]. It has been speculated that the differences in clinicopathological features between BSNS and alveolar RMS are probably determined by the cell of origin and cellular environment and by additional genetic aberrations [4].

Nevertheless, the case we are presenting herein shows that a very small subset of BSNS may progress towards a high-grade RMS. After review of the literature, we found 2 recently reported cases of molecularly confirmed BSNS with high-grade transformation, their clinicopathological features together with our case are summarized in Table 1. From the available description and figures, it seems that both cases showed a high-grade spindle cell morphology without the rhabdoid features detected in our case. Unfortunately, the first case was not tested for skeletal muscle markers at all [14], and the other was tested only for desmin and myogenin, both of which were focally positive in the high-grade areas of this case, suggesting differentiation into spindle cell RMS [15].

**Table 1.** Clinicopathological features of BSNS cases with high-grade progression

	Case 1 (Bell D et al^11^)	Case 2 (Hasnie S et al^12^)	Current case
Age/sex	66/M	72/F	67/M
Size (cm)	3.0 × 2.4 × 2.0	NA	4.4 x 3.4 x 2.2
Course of disease	BSNS recurring as high-grade sarcoma in superior extraconal orbit with intracranial extension 15 years later.	2-year history of progressive nasal obstruction with epistaxis and headaches. Resection of a “polyp” which was diagnosed as BSNS. Then resection of a skull base lesion encompassing the entirety of both sinonasal cavities.	History of septoplasty, bilateral frontal sinusotomy, and removal of right middle turbinate concha bullosa 3 years before presenting with nasal congestion and epiphora. MRI showed a soft tissue mass involving the right ethmoid, maxillary, and frontal sinuses and invading the extraconal orbit causing mild asymmetric right-sided ocular proptosis
Treatment and outcome	Primary tumor – resection, radiotherapy, chemotherapy. Recurrence – resection, irradiation. No evidence of disease (10 months)	Resection. Died on complications related to surgery 4.5 months after the resection with no signs of tumor recurrence	Neoadjuvant chemotherapy, resection, irradiation. Died 15 months after diagnosis on tumor progression
Morphology	High-grade spindle cell sarcoma	High-grade spindle cell sarcoma	High-grade rhabdomyosarcoma
IHC of the high-grade areas	Patchy SMA and S100 expression, myogenic markers not tested	Focal desmin and myogenin	Desmin, PAX7, MyoD1, Myogenin positive
Molecular genetics	*PAX3::MAML3* fusion	*PAX3* gene break, copy number alterations of 9p and 22	*PAX3::MAML3* fusion

The differential diagnosis of ordinary BSNS includes a list of other neoplasms with uniform spindle cell morphology occurring in this anatomic area such as malignant peripheral nerve sheath tumor (including malignant Triton tumor when rhabdomyoblastic differentiation is present), cellular schwannoma, monophasic fibrous synovial sarcoma, sinonasal glomangiopericytoma, and solitary fibrous tumor. However, the distinction is usually possible using a carefully selected panel of immunohistochemical stains [1–4]. In contrast, the rhabdomyosarcomatous component in our case could be mistaken for embryonal RMS or pleomorphic RMS. If this area is sampled without the conventional BSNS component (as happened in the initial probatory biopsy in our case), the distinction is impossible without molecular genetic methods. Since the clinical behavior and/or response to treatment of embryonal and pleomorphic RMS might differ from RMS arising from BSNS, we believe it is reasonable that molecular investigation of such cases in the sinonasal area with either *PAX3* FISH probe or preferably using an adequate RNA-sequencing panel is carried out. Significant prognostic differences have already been noted between several molecular subgroups of spindle cell RMS. For example, spindle cell RMS with *VGLL2* and *VGLL3* fusions has a relatively favorable prognosis which is in contrast to the very aggressive subset harboring *MYOD1* mutations [16]. Importantly, spindle cell RMS with *VGLL3* fusions and *MYOD1* mutations has a predilection for the head and neck area [16]. As the other published case of BSNS with high-grade transformation featured spindle cell RMS pattern, a comprehensive molecular investigation for this morphological variant of RMS is warranted as well. Lastly, the herein presented case emphasizes the importance of careful follow-up of patients with BSNS and a throughout sampling of every case to prevent a delayed detection of high-grade transformation.

With regard to the case presented herein, it is interesting to note the significantly different expression of skeletal muscle markers in the conventional BSNS areas compared to the high-grade areas. Even though the former areas were diffusely positive for PAX7 and MyoD1, they were completely negative for desmin and myogenin. In contrast, the high-grade areas were moderately to diffusely positive for all these markers. Both PAX7 and MyoD1 regulate earlier stages of mammalian myogenesis than myogenin [17], and all these 3 transcriptional factors are expressed significantly earlier in myogenesis than the structural protein desmin [18]. This suggests that the cells in conventional BSNS areas were arrested at earlier stages of myogenesis which would be also in line with a generally more frequent expression of MyoD1 compared to the expression of desmin and myogenin in the previous studies [3, 4]. In contrast, possibly due to additional molecular aberrations, the cells in the high-grade areas, although being highly anaplastic, were apparently able to differentiate further along the myogenic pathway as also evidenced by their rhabdoid morphology.

In conclusion, we presented a unique case of BSNS with transformation into high-grade RMS which together with 2 previously published cases of BSNS with high-grade transformation helps to better understand this novel phenomenon. Although the risk for high-grade transformation of BSNS appears low, it has important clinical and diagnostic implications. Besides advocating for a careful follow-up of patients with BSNS and a throughout sampling of every case, we believe that molecular profiling of sinonasal RMS of any type is warranted.

## References

[1] Lewis JT, Oliveira AM, Nascimento AG, Schembri-Wismayer D, Moore EA, Olsen KD (2012). Low-grade sinonasal sarcoma with neural and myogenic features: a clinicopathologic analysis of 28 cases. Am J Surg Pathol..

[2] Carter CS, East EG, McHugh JB (2018). Biphenotypic sinonasal sarcoma: a review and update. Arch Pathol Lab Med..

[3] Le Loarer F, Laffont S, Lesluyes T, Tirode F, Antonescu C, Baglin AC (2019). Clinicopathologic and molecular features of a series of 41 biphenotypic sinonasal sarcomas expanding their molecular spectrum. Am J Surg Pathol..

[4] Fritchie KJ, Jin L, Wang X, Graham RP, Torbenson MS, Lewis JE (2016). Fusion gene profile of biphenotypic sinonasal sarcoma: an analysis of 44 cases. Histopathology..

[5] Huang SC, Ghossein RA, Bishop JA, Zhang L, Chen TC, Huang HY (2016). Novel PAX3-NCOA1 fusions in biphenotypic sinonasal sarcoma with focal rhabdomyoblastic differentiation. Am J Surg Pathol..

[6] Wang X, Bledsoe KL, Graham RP, Asmann YW, Viswanatha DS, Lewis JE (2014). Recurrent PAX3-MAML3 fusion in biphenotypic sinonasal sarcoma. Nat Genet..

[7] Michal M, Rubin BP, Kazakov DV, Michalova K, Steiner P, Grossmann P (2020). Inflammatory leiomyosarcoma shows frequent co-expression of smooth and skeletal muscle markers supporting a primitive myogenic phenotype: a report of 9 cases with a proposal for reclassification as low-grade inflammatory myogenic tumor. Virchows Arch..

[8] Georgantzoglou N, Green D, Stephen SA, Kerr DA, Linos K (2022). Biphenotypic sinonasal sarcoma with PAX7 expression. Int J Surg Pathol..

[9] Nichols MM, Alruwaii F, Chaaban M, Cheng YW, Griffith CC (2022) Biphenotypic sinonasal sarcoma with a novel PAX3::FOXO6 fusion: a case report and review of the literature. Head Neck Pathol. 10.1007/s12105-022-01479-w 10.1007/s12105-022-01479-w PMC10063736 36169791

[10] Mechtersheimer G, Andrulis M, Delank KW, Volckmar AL, Zhang L, von Winterfeld M (2021). RREB1-MKL2 fusion in a spindle cell sinonasal sarcoma: biphenotypic sinonasal sarcoma or ectomesenchymal chondromyxoid tumor in an unusual site?. Genes Chromosom. Cancer..

[11] Siegfried A, Romary C, Escudie F, Nicaise Y, Grand D, Rochaix P (2018). RREB1-MKL2 fusion in biphenotypic "oropharyngeal" sarcoma: new entity or part of the spectrum of biphenotypic sinonasal sarcomas?. Genes Chromosom. Cancer.

[12] Agaimy A, Din NU, Dermawan JK, Haller F, Melzer K, Denz A (2023). RREB1::MRTFB fusion-positive extra-glossal mesenchymal neoplasms: a series of five cases expanding their anatomic distribution and highlighting significant morphological and phenotypic diversity. Genes Chromosom. Cancer.

[13] Buckingham M, Relaix F (2007). The role of Pax genes in the development of tissues and organs: Pax3 and Pax7 regulate muscle progenitor cell functions. Annu Rev Cell Dev Biol..

[14] Bell D, Phan J, DeMonte F, Hanna EY (2022). High-grade transformation of low-grade biphenotypic sinonasal sarcoma: radiological, morphophenotypic variation and confirmatory molecular analysis. Ann Diagn Pathol..

[15] Hasnie S, Glenn C, Peterson JEG, El Rassi ET, McKinney KA (2022). High-grade biphenotypic sinonasal sarcoma: a case report. J Neurol Surg Rep..

[16] Agaimy A, Dermawan JK, Leong I, Stoehr R, Swanson D, Weinreb I (2022). Recurrent VGLL3 fusions define a distinctive subset of spindle cell rhabdomyosarcoma with an indolent clinical course and striking predilection for the head and neck. Genes Chromosom. Cancer.

[17] Charville GW, Varma S, Forgo E, Dumont SN, Zambrano E, Trent JC (2016). PAX7 Expression in rhabdomyosarcoma, related soft tissue tumors, and small round blue cell neoplasms. Am J Surg Pathol..

[18] Folpe AL (2002). MyoD1 and myogenin expression in human neoplasia: a review and update. Adv Anat Pathol..

